# Comparison of oliceridine and sufentanil in postoperative pain management for postoperative nausea and vomiting after bimaxillary orthognathic surgery: an exploratory randomized controlled trial

**DOI:** 10.3389/fmed.2026.1839035

**Published:** 2026-06-05

**Authors:** Yi Zhou, Guoli Xiong, Likuan Wang, Yun Liu, Xiaodong Wang, Xudong Yang

**Affiliations:** 1 Department of Anesthesiology, Peking University School and Hospital of Stomatology, Beijing, China; 2 National Center of Stomatology and National Clinical Research Center for Oral Diseases, Beijing, China

**Keywords:** bimaxillary orthognathic surgery, oliceridine, PCIA, PONV, sufentanil

## Abstract

**Background:**

Postoperative nausea and vomiting (PONV) is a prevalent adverse event following bimaxillary orthognathic surgery, with opioids being a significant contributing factor. Oliceridine, a G protein-biased *μ*-opioid receptor agonist, reduces activation of the *β*-arrestin pathway and may decrease gastrointestinal adverse events relative to traditional opioids. This study aimed to evaluate the efficacy and safety of oliceridine relative to sufentanil for postoperative patient-controlled intravenous analgesia (PCIA) in this population, with a particular emphasis on PONV.

**Methods:**

This prospective, double-blind, randomized controlled exploratory trial was conducted at the Peking University School and Hospital of Stomatology from March to August 2025. A total of 100 eligible patients (ASAI-II, BMI 18–24 kg/m^2^, aged 18–60 years) were randomly assigned in a 1:1 ratio to receive either oliceridine (0.25 mg/kg combined with tropisetron 10 mg, designated as Group O) or sufentanil (1.25 μg/kg combined with tropisetron 10 mg, designated as Group S) via patient-controlled intravenous analgesia (PCIA), which included a 2 mL/h background infusion, 0.5 mL bolus, 15-min lockout interval. All participants were administered multimodal antiemetic prophylaxis and standardized general anesthesia. The primary outcome measure was the incidence of PONV within 72 h. Secondary outcome measures included the severity of PONV, the use of rescue antiemetics, postoperative pain scores assessed using an 11-point numerical rating scale (NRS), and the occurrence of adverse events. Statistical analyses were conducted using SPSS version 26.0, with a significance threshold set at *p* < 0.05.

**Results:**

The baseline characteristics were comparable between the groups. The incidence of PONV within 72 h was lower in Group O compared to Group S (40.0% vs. 50.0%; odds ratio [OR] = 0.67, 95% confidence interval [CI]: 0.30–1.47; *p =* 0.315). Similarly, the incidences of moderate-to-severe PONV (26.0% vs. 36.0%; OR = 0.59, 95% CI: 0.28–1.25; *p* = 0.108) and the requirement for rescue antiemetics (20.0% vs. 34.0%; OR = 0.49, 95% CI: 0.20–1.20; *p =* 0.115) were lower in Group O. However, none of these differences reached statistically significance. A repeated-measures analysis of variance (ANOVA) for moderate-to-severe postoperative pain revealed a significant effect of time (*F* = 17.189, *p* < 0.001) but no significant group-time interaction (*p* = 0.142). No serious adverse events, such as respiratory depression or hypotension, were reported in either group, and the incidence of dizziness was similar between the groups (22% vs. 24%, *p* > 0.05).

**Conclusion:**

Oliceridine demonstrated comparable analgesic efficacy and safety to sufentanil, exhibiting numerically lower, albeit not statistically significant, differences in postoperative nausea and vomiting (PONV) and the use of rescue antiemetics. Considering the exploratory nature of this study and the determination of the sample size based primarily on clinical feasibility rather than statistical power, these findings should be regarded as preliminary. Further validation in larger, adequately powered trials incorporating dose-optimization designs is necessary.

**Clinical trial registration:**

https://www.chictr.org.cn/showproj.html?proj=257554, Identifier: ChiCTR2500096345.

## Introduction

Postoperative nausea and vomiting (PONV) is a frequently encountered adverse event following surgical procedures, affecting approximately 30% of all surgical patients (1). Orthognathic surgery, which involves maxillary and/or mandibular osteotomies for the correction of dentofacial deformities, is associated with a high incidence of PONV (2–4). A previous prospective study conducted at our institution revealed that patients undergoing bimaxillary surgery experienced a PONV incidence rate of up to 61.0% when postoperative analgesia included the opioid sufentanil (5).

The impact of PONV on recovery is complex and multifaceted. PONV can impede the resumption of oral intake, leading to potential electrolyte imbalances. Frequent vomiting may also contribute to complications such as wound bleeding or dehiscence and heightens the risk of aspiration. Furthermore, PONV is linked to prolonged stays in the Post Anesthesia Care Unit (PACU), extended hospitalizations, and an increased medical burden on patients (6). Advances in anesthetic drugs and techniques have significantly enhanced the safety of anesthesia, shifting the focus toward the quality of anesthesia care, with perioperative patient satisfaction being a primary concern. Research indicates that PONV is among the most distressing postoperative complications for surgical patients (7, 8), and there is a notable correlation between decreased patient satisfaction with surgical anesthesia and the occurrence of PONV (9). In patients undergoing orthognathic surgery, PONV can more severely impact postoperative recovery by exacerbating intraoral bleeding, with swallowed blood potentially prolonging PONV. Additionally, limited mouth opening or maxillomandibular elastic traction during the early postoperative period may increase the risk of choking during episodes of vomiting.

Opioids are extensively utilized perioperatively for pain management and constitute an independent risk factor for PONV (10). The precise mechanisms by which opioids elicit nausea and vomiting are not fully elucidated, but they likely involve multiple intricate pathways. The Chemoreceptor Trigger Zone (CTZ), situated in the Area Postrema (AP) near the floor of the fourth ventricle, is rich in opioid receptors, predominantly mu (*μ*) and delta (*δ*)-opioid receptors. Activation of these receptors by opioids transmits signals to the Vomiting Centre, thereby initiating the vomiting reflex (11–13). Additionally, opioids may directly stimulate the vestibular apparatus and activate gastrointestinal opioid receptors, resulting in uncoordinated intestinal activity, impaired gastrointestinal motility, intestinal distension, and spasms, all of which may contribute to nausea and/or vomiting (13). Evidence suggests that intraoperative opioid use administration does not serve as a sustained stimulus for PONV (10). Conversely, the use of opioids postoperatively for pain management can provoke PONV, with the risk escalating in a dose-dependent manner (14–16).

Traditional opioid agonists interact with *μ*-opioid receptors, activating the G protein signaling pathway to induce analgesic effects. However, they concurrently activate the *β*-arrestin protein pathway, which is associated with respiratory depression and gastrointestinal-related adverse effects (17). In contrast, oliceridine is an innovative intravenous opioid that selectively activates the G protein pathway without engaging the *β*-arrestin pathway, thereby reducing the incidence of drug-related adverse effects (18, 19).

Previous research (20, 21) has indicated that patients administered postoperative oliceridine at doses equianalgesic to morphine exhibit a reduced incidence of gastrointestinal adverse events such as nausea or vomiting. A recently published randomized controlled trail (22) further demonstrated that oliceridine, when used for postoperative analgesia significantly decreased the 48-h incidence of PONV compared to sufentanil (32.3% vs. 50.8%, *p* = 0.033). Additionally, the incidence of moderate-to-severe PONV (18.5% vs. 38.5%, *p* = 0.012) was significantly lower in the oliceridine group, while maintaining comparable analgesic efficacy between the two groups.

The primary objective of this study was to evaluate the efficacy of oliceridine, in comparison to sufentanil, in reducing the incidence and severity of postoperative nausea and vomiting in patients receiving postoperative analgesia following bimaxillary orthognathic surgery.

## Materials and methods

### Study design and participants

This prospective, double -blind, randomized controlled trial was undertaken following approval from the Ethics Committee of Peking University School and Hospital of Stomatology (PKUSSIRB-2024104172) and was registered with the Chinese Clinical Trial Registry (ID: ChiCTR2500096345) on January 18, 2025. The study adhered to the ethical principles delineated in the Declaration of Helsinki, and written informed consent was obtained from all participants.

The study was conducted from March 2025 to August 2025 at the Peking University School and Hospital of Stomatology in Beijing, China. The inclusion criteria encompassed adult patients aged 18 to 59 years, classified as American Society of Anesthesiologists (ASA) class I or II, with a body mass index (BMI) ranging from 18 to 23.9 kg/m^2^. These patients were scheduled to undergo elective bimaxillary orthognathic surgery and required postoperative analgesia via a pump. Exclusion criteria included: 1) pregnant or lactating women; 2) individuals with a history of alcoholism, opioid dependence, or other substance abuse disorders; 3) preoperative use of CYP2D6 inhibitors (such as paroxetine or fluoxetine); 4) a history of motion sickness or the occurrence of vomiting or retching within 24 h prior to surgery; 5) participation in another clinical trial within 3 months prior to enrollment; and 6) refusal to participate in the study.

### Randomization, blinding, and interventions

Participants were randomly assigned to one of two groups: the oliceridine PCIA group (Group O) or the sufentanil PCIA group (Group S). Random numbers were generated using SPSS software in a 1:1 ratio and were placed in sequentially numbered opaque envelopes. These envelopes were opened prior to the end of the surgery by an anesthesia nurse responsible for preparing the patient-controlled intravenous analgesia (PCIA) pump; this nurse did not participate in any other aspect of the study. The PCIA regimen consisted of either oliceridine (Jiangsu Nhwa Pharmaceutical Co., Ltd., China) at a dose of 0.25 mg/kg (20, 21) or sufentanil (Humanwell Healthcare Co., Ltd., China) at a dose of 1.25 μg/kg (5), both combined with tropisetron (10 mg) and diluted with normal saline to a total volume of 100 mL. The PCIA pump parameters were set at a continuous background infusion of 2 mL/h, a bolus dose of 0.5 mL, and a lock-out interval of 15 min. The envelopes were resealed after the preparation of the PCIA pump and remained closed until the end of the trial. As a result, care providers, outcome assessors and patients were blinded to study group assignment.

### Anesthesia management and perioperative care

Intraoperative monitoring encompassed a 3-lead electrocardiogram, invasive blood pressure measurement via the dorsalis pedis artery, pulse oximetry, nd-tidal carbon dioxide concentration, inhalational anesthetic concentration, inhalational anesthetic concentration, bispectral index (BIS), and body temperature.

All patients were subjected to a standardized general anesthesia protocol, without the administration of premedication. Vascular access was established in the operating room. Prior to induction, each patient received 10 mg of dexamethasone, and intravenous midazolam was administered at the discretion of the attending anesthesiologist. Anesthesia induction was achieved using either propofol or etomidate, along with sufentanil, and either rocuronium or cisatracurium, followed by nasotracheal intubation. Maintenance of anesthesia was accomplished through a continuous intravenous infusion of propofol in combination with remifentanil, supplemented by intermittent boluses of sufentanil as necessary. This regimen was optionally augmented with inhalational sevoflurane or a dexmedetomidine infusion, based on the attending anesthesiologist’s judgment. Mechanical ventilation was sustained using an oxygen-air mixture. Vasoactive agents were employed to ensure hemodynamic stability or to induce controlled hypotension when indicated. Upon completion of the surgical procedure, opioids were administered as required; subsequently, all patients received 2 mg of tropisetron and 100 mg of flurbiprofen, and a pre-prepared PCIA pump was initiated.

Postoperatively, patients were transferred to the post-anesthesia care unit (PACU) while maintaining nasotracheal intubation. Sedation with dexmedetomidine was continued, and flurbiprofen and/or opioids were administered based on clinical necessity. Extubation was conducted once patients satisfied established criteria, including regained consciousness, complete neuromuscular blockade recovery, intact protective airway reflexes, and hemodynamic stability. The decision to discharge patients from the PACU to the general ward was made by the attending anesthesiologist, typically occurring the following morning. Rescue antiemetics, such as metoclopramide and/or tropisetro, were prescribed as required by the attending anesthesiologist or surgeon.

### Data collection

The baseline data comprised demographic characteristics, surgical diagnoses, preoperative comorbidities—including the Charlson Comorbidity Index—smoking history, and Apfel’s score (23). Intraoperative dataincluded the duration of anesthesia, the types and dosages of anesthetics and adjunctive medications, the type and duration of surgery, and fluid balance.

Patients were assessed for postoperative nausea and vomiting (PONV) and pain at 6, 12, 24, 48, and 72 h following surgery. Nausea was identified through direct questioning, and its severity was measured using an 11-point Numerical Rating Scale (NRS), where scores ranged from 0, indicating no nausea, to 10, indicating the worst possible nausea. Vomiting was characterized by episodes of retching or the expulsion of intragastric contents. Pain severity was similarly assessed using the 11-point NRS, with 0 representing no pain and 10 denoting the worst possible pain. Furthermore, the incidence of adverse reactions, including dizziness, respiratory depression, constipation, and urinary retention, was documented within the 72-h postoperative period.

### Outcomes

The primary outcome was the incidence of PONV, defined as the occurrence of any nausea, retching, or vomiting within 72 h following surgery. Secondary outcomes were as follows: (1) The severity of PONV within the 72 h postoperative period, assessed using the Numeric Rating Scale (NRS, 0–10) for nausea and by documenting episodes of vomiting or retching, with specific definitions provided in Table 1 (5). (2) Incidence of moderate-to-severe PONV across various postoperative time intervals (0–6 h, > 6–12 h, > 12–24 h, > 24–48 h, and > 48–72 h). (3) The utilization of rescue antiemetics within 72 h post-surgery. Additional outcomes included: (1) Postoperative pain scores across different postoperative periods. (2) The incidence of adverse events within 72 h after surgery.

**Table 1:** The severity of PONV.

Severity	Definition
No	No nausea or emetic symptoms
Mild	Mild nausea (NRS 1–3) or one episode vomiting if caused by an exogenous stimulus
Moderate	Two times vomiting, or experienced nausea that required a rescue antiemetic therapy only once
Severe	More than two emetic episodes or necessitating more than one dose of a rescue antiemetic

### Statistical analysis

The study aimed to enroll a total of 100 eligible patients, were planned for enrollment in this study, with an equal allocation of 50 patients to Group O and 50 to Group S. The determination of the sample size was guided by the exploratory nature of the research, taking into account the feasibility of clinical recruitment and the necessity to generate preliminary evidence to inform future confirmatory studies.

All statistical analyses were were conducted utilizing SPSS Statistics version 26.0 (IBM Corp., Armonk, NY, United States). Continuous variables adhering to a normal distribution were reported as mean ± standard deviation (Mean ± SD), with intergroup comparisons executed via the independent-samples t-test. Continuous variables that did not conform to a normal distribution were presented as median and interquartile range [M (Q1, Q3)], with intergroup comparisons performed using the Mann–Whitney U test. Categorical variables were described in terms of frequency and percentage [*n* (%)]. For unordered categorical variables, intergroup comparisons were conducted using the Pearson *χ^2^* test or Fisher’s exact test when expected frequencies were less than 5. Ordered categorical variables were compared between groups utilizing the Mann–Whitney *U* test. Repeated-measures analysis of variance (ANOVA) was employed to evaluate differences in postoperative nausea and vomiting (PONV) severity and pain scores across different time points. All hypothesis tests were two-tailed, with a predefined significance threshold of *α* = 0.05. A *p*-value of less than 0.05 was considered indicative of statistical significance.

## Results

### Baseline characteristics and perioperative data

**Figure 1:**
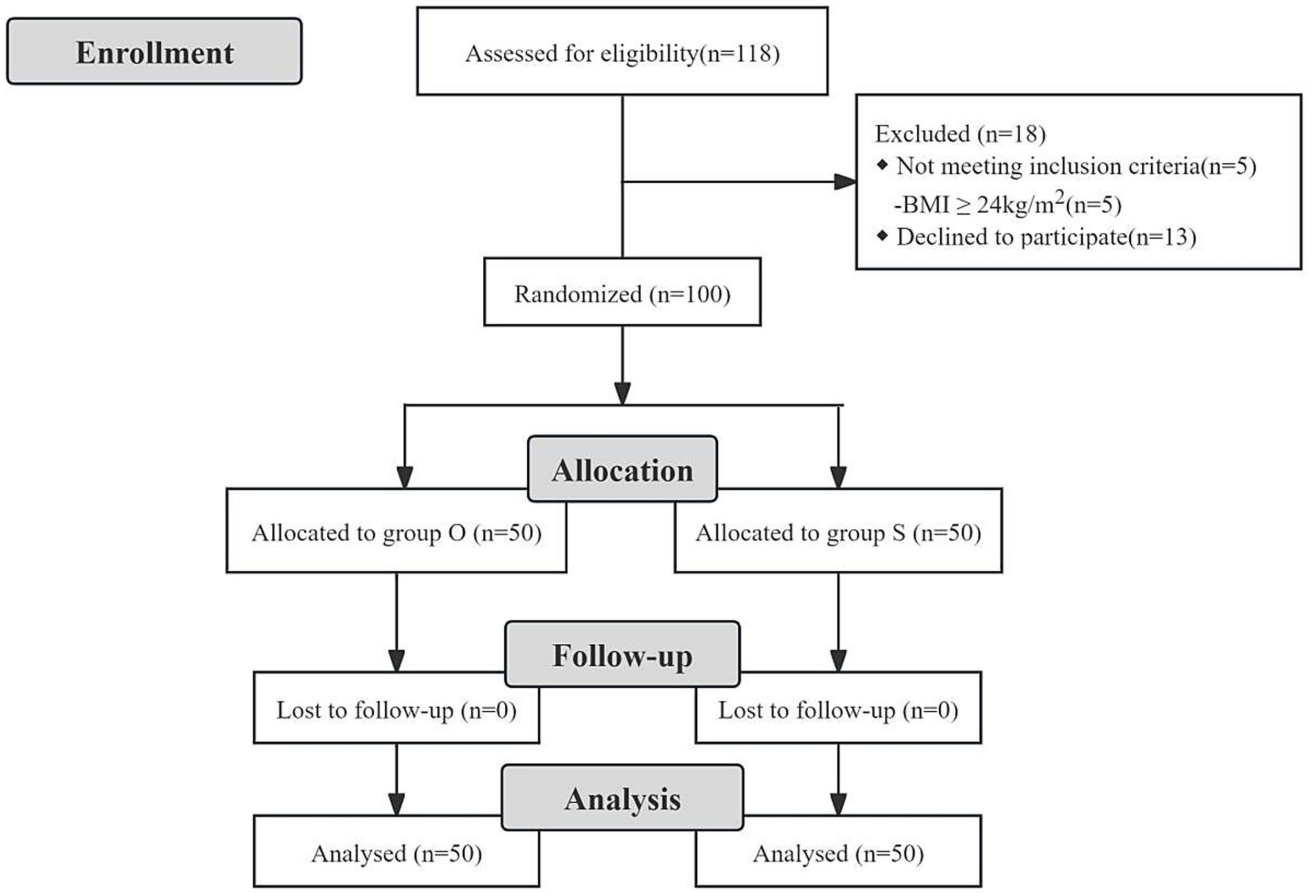
Consolidated standards of reporting trials diagram (CONSORT flow diagram).

Between March 2025 and August 2025, a total of 118 patients were initially evaluated for inclusion in the study. Of these, 13 patients declined participation, and an additional 5 did not meet the inclusion criteria. Consequently, 100 patients provided consent to participate in the study. No patients were lost to follow-up during hospitalization (see Figure 1). While baseline characteristics were generally well balanced between the groups, a minor difference was observed in the ASA classification: four patients (8%) in Group O were classified as ASA II, whereas all patients in Group S were classified as ASA I (*p* = 0.126) (refer to Table 2). Intraoperative and postoperative data were similarly comparable between the two groups (see Table 3). A multivariable logistic regression analysis was conducted, incorporating intraoperative sevoflurane use, intraoperative dexmedetomidine use, intraoperative sufentanil dose, and intraoperative remifentanil dose as independent variables, with the primary outcome as the dependent variable. The analysis revealed that none of these factors were significantly associated with the risk of 72-h postoperative nausea and vomiting (PONV) (see Table 4).

**Table 2:** Demographic and baseline data of two groups of patients.

Variable	Group O (*n* = 50)	Group S (*n* = 50)	*p*-value
Age (y)	25.82 ± 5.06	24.82 ± 4.76	0.311
Sex			0.181
Male	17 (34.00)	11 (22.00)	
Female	33 (66.00)	39 (78.00)	
BMI(kg/m^2^)	21.09 ± 1.73	21.21 ± 1.84	0.729
ASA classifcation			0.126
I	46 (92.00)	50 (100.00)	
II	4 (8.00)	0 (0.00)	
Preoperative comorbidities			
Obstructive sleep apnea^a^	3 (6.00)	3 (6.00)	1.000
Hyperthyroidism	0(0.00)	0(0.00)	1.000
Hypothyroidism	0(0.00)	0(0.00)	1.000
Hepatitis B	3 (6.00)	0 (0.00)	0.241
Hypertension	0(0.00)	0(0.00)	1.000
Charlson comorbidity index			0.241
0	47 (94.00)	50 (100.00)	
1	3 (6.00)	0 (0.00)	
No smoking history^b^	49 (98.00)	49 (98.00)	1.000
Previous surgery	8 (16.00)	7 (14.00)	0.779
Apfel risk factors			0.194
1	1 (2.00)	1 (2.00)	
2	16 (32.00)	10 (20.00)	
3	33 (66.00)	39 (78.00)	
4	0(0.00)	0(0.00)	

Data are mean±SD, median (IQR), or *n* (%).

ASA, American Society of Anesthesiologists; Body Mass Index;

Group O, the oliceridine PCIA group; Group S, the sufentanil PCIA group.

^a^Diagnosed with polysomnography.

^b^Smoking history was defined as those who have smoked more than 100 cigarettes in their lifetime.

**Table 3:** Intra and postoperative data of two groups of patients.

Variable	Group O (*n* = 50)	Group S (*n* = 50)	*p*-value
Intraoperative data			
Duration of anesthesia (min)	250.14 ± 42.22	259.82 ± 54.51	0.323
Duration of operation (min)	192.62 ± 39.00	209.14 ± 52.81	0.078
Additional procedures			
Segmental osteotomy	13 (26.00)	13 (26.00)	1.000
Subapical osteotomy	2 (4.00)	3 (6.00)	1.000
Genioplasty	41 (82.00)	33 (66.00)	0.068
Intraoperative medications			
Sevofurane	41 (82.00)	45 (90.00)	0.249
Dose of midazolam (mg)	0.00 (0.00, 2.38)	0.00 (0.00, 2.00)	0.731
Dose of propofol (mg/kg)	24.74 (17.95, 28.63)	24.43 (19.30, 28.25)	0.92
Etomidate	18 (36.00)	17 (34.00)	0.834
Dose of etomidate	0.00 (0.00, 12.00)	0.00 (0.00, 11.50)	0.811
Dose of sufentanil (μg/kg)	0.63 (0.50, 0.78)	0.67 (0.55, 0.82)	0.478
Dose of remifentanil (μg/kg)	33.33 (27.48, 43.45)	34.82 (29.82, 40.00)	0.699
Dexmedetomidine	8 (16.00)	9 (18.00)	0.790
Dose of dexmedetomidine (μg/kg)	0.00 (0.00, 0.00)	0.00 (0.00, 0.00)	0.780
Antihypertensive drugs			
Nicardipine	23 (46.00)	19 (38.00)	0.418
Urapidil	2 (4.00)	2 (4.00)	1.000
Esmolol	17 (34.00)	19 (38.00)	0.677
Duration of Controlled hypotension(min)	70.00 (45.00, 100.00)	73.50 (35.00, 110.00)	0.841
Intravenous fuid (ml)	1582.00 ± 201.72	1642.00 ± 290.73	0.234
Infusion of hydroxyethyl starch	8 (16.00)	5 (10.00)	0.372
Estimated blood loss (ml)	200.00 (150.00, 200.00)	200.00 (150.00, 200.00)	0.663
Urine output (ml)	300.00 (200.00, 437.50)	300.00 (150.00, 500.00)	0.912
Postoperative data			
Duration in PACU^a^ (min)	650.00 (281.25, 911.25)	682.50 (294.00, 834.50)	0.535
Time to extubation (min)	90.00 (60.00, 120.00)	90.00 (65.00, 120.00)	0.681
Use of dexmedetomidine in PACU	36 (72.00)	31 (62.00)	0.288
Dose of dexmedetomidine in PACU (μg/kg)	0.74 (0.00, 1.31)	0.55 (0.00, 1.57)	0.749
Intravenous fuid in PACU (ml)	1619.00 ± 510.89	1598.00 ± 441.70	0.826
Urine output in PACU (ml)	750.00 (300.00, 1047.50)	600.00 (300.00, 900.00)	0.597
Drainage in PACU (ml)	60.00 (40.00, 77.50)	55.00 (50.00, 95.00)	0.610
Medication during 72 h after surgery			
Total sufentanil equivalent dose (μg)	77.52 ± 10.45	73.64 ± 10.63	0.069
Total sufentanil equivalent dose (μg/kg)	1.28 ± 0.05	1.27 ± 0.04	0.374
Non-steroidal anti-infammatory drugs			
Flurbiprofen axetil	16 (32.00)	13 (26.00)	0.509
Loxoprofen	1 (2.00)	0 (0.00)	1.000

Data are mean±SD, median (IQR), or *n* (%).

PACU, post-anesthesia care unit.

Sufentanil equivalent dose consumed within 72 h after surgery (including postoperative analgesia pump).

**Table 4:** Multivariable logistic regression analysis of factors associated with 72 h PONV.

Variable	B	SE	z	Wald *χ^2^*	*p-*value	OR (5% CI)
Sevoflurane	−0.680	0.595	−1.143	1.307	0.253	0.506 (0.158, 1.626)
Dexmedetomidine	0.524	0.571	0.918	0.843	0.359	1.690 (0.551, 5.177)
Sufentanil dose (ug/kg)	0.670	0.681	0.983	0.967	0.325	1.955 (0.514, 7.432)
Remifentanil dose (ug/kg)	−0.001	0.018	−0.059	0.003	0.953	0.999 (0.965, 1.034)

OR, odds ratio; CI, confidence interval; SE, standard error.

### Primary outcome

PONV occurred in 40.0% (20 out of50) of patients in Group O and 50.0% (25 out of 50) of patients in the Group S within 72 h. The difference between the two groups was not statistically significant (OR = 0.67, 95% CI: 0.30–1.47; *p* = 0.315).

### Secondary outcomes

The severity of PONV within 72 h did not did not exhibit a statistically significant difference between the two groups (OR = 0.59, 95%CI: 0.28–1.25; *p* = 0.108). The incidence of moderate-to-severe PONV was observed to be 26.0% in Group O compared to 36.0% in Group S. Regarding the requirements for rescue antiemetic therapy within the 72-h period, 20.0% (10 out of 50) of patients in Group O required such intervention, with 2 patients necessitating a second dose. Conversely, 34.0% (17 out of 50) of patients in Group S required rescue antiemetics, with 8 patients receiving a second administration. However, the difference in the requirement for rescue antiemetic therapy between the two groups was not statistically significant (OR = 0.49, 95%CI: 0.20–1.20; *p* = 0.115) (Table 5).

**Table 5:** The incidence of PONV and use of rescue antiemetics of two groups of patients.

Variable	Group O (*n* = 50)	Group S (*n* = 50)	*p*-value	OR (95%CI)
Primary outcome				
PONV within 72 h	20 (40.00)	25 (50.00)	0.315	0.67 (0.30, 1.47)
Secondary outcomes				
Severity of PONV			0.108	0.59 (0.28, 1.25)
None	30(60.00)	25(50.00)		
Mild	7 (14.00)	7 (14.00)		
Moderate	8 (16.00)	5 (10.00)		
Severe	5 (10.00)	13 (26.00)		
Rescue				
Antiemetics within 72 h	10 (20.00)	17 (34.00)	0.115	0.49 (0.20, 1.20)
Rescue times			0.079	0.45 (0.18, 1.10)
0	40 (80.00)	33 (66.00)		
1	8 (16.00)	9 (18.00)		
2	2 (4.00)	8 (16.00)		

Data are *n* (%), OR, odds ratio; CI, confidence interval.

**Figure 2:**
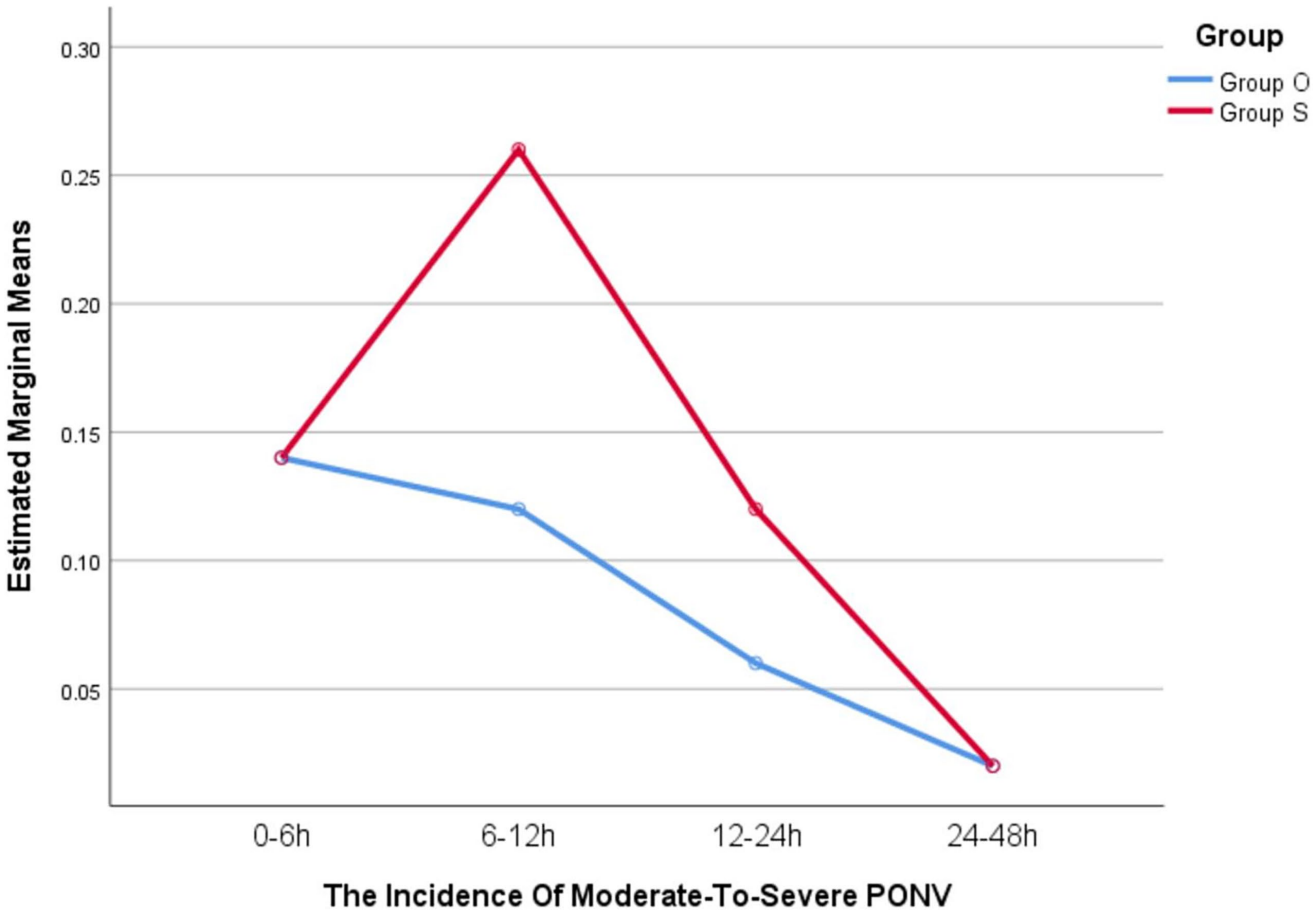
Estimated marginal means of the moderate-to-severe PONV incidence in the two groups at four postoperative time points.

**Figure 3:**
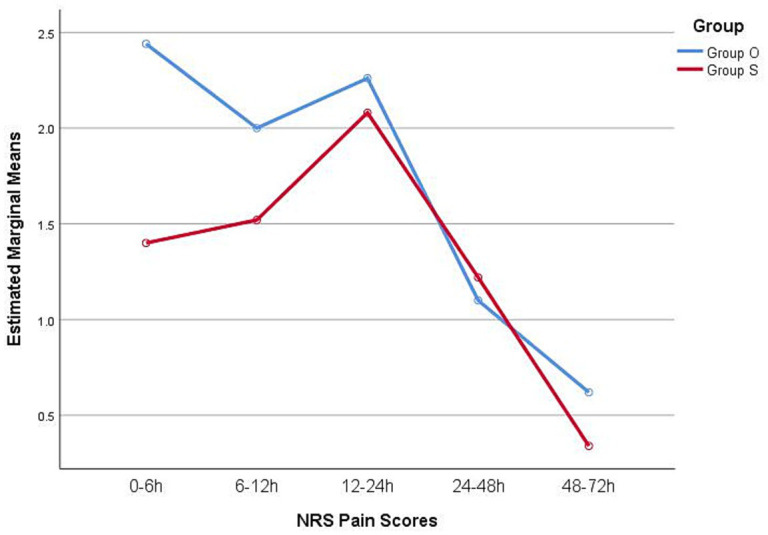
Estimated marginal means of the NRS pain scores in the two groups at four postoperative time points.

A repeated-measures ANOVA was conducted to assess the incidence of moderate-to-severe PONV across different postoperative time between the two groups. The analysis revealed that the temporal trends of PONV did not differ significantly between the groups (*F* = 1.401, *p* = 0.243, partial η^2^ = 0.014, 95% CI: 0.003, 0.061). However, a significant main effect of time was observed (*F* = 6.706, *p* < 0.001, partial η^2^ = 0.088, 95% CI: 0.051, 0.153), indicating a significant change in the incidence of moderate-to-severe PONV over time, characterized by an overall downward trend (see Figure 2). At 48–72 h postoperatively, the incidence of moderate-to-severe PONV was zero in both groups; consequently, this time point was excluded from the analysis. A repeated-measures ANOVA conducted on the incidence of moderate-to-severe postoperative pain revealed that the interaction effect between group and time did not achieve statistical significance (*F* = 1.732, *p* = 0.142, partial η^2^ = 0.001, 95% CI: 0.000, 0.052). However, the incidence of moderate-to-severe pain demonstrated significant variation over time (*F* = 17.189, *p* < 0.001, partial η^2^ = 0.016, 95% CI [0.004, 0.064]) (refer to Figure 3). Bonferroni-corrected *post hoc* analyses indicated a significant reduction in the incidence of moderate-to-severe PONV was reduced at 24–48 h postoperatively, compared to the intervals of 0–6 h and 6–12 h (*p* = 0.007 and *p* = 0.001, respectively). Regarding moderate-to-severe pain, the incidence demonstrated a progressive decline over time, with significantly lower rates observed at 48–72 h compared to the preceding four intervals, and at 24–48 h compared to 12–24 h (all *p* < 0.001).

Table 6 presents a comparative analysis of adverse events occurring within 72 h postoperatively between Group O (*n* = 50) and Group S (*n* = 50). The analysis revealed no statistically significant differences in the incidence of adverse events between the two groups. Furthermore, neither group experienced cases of respiratory depression, hypotension, urinary retention, or constipation. The incidence of dizziness was reported at 22% in Group O and 24% in Group S, with the difference not reaching statistical significance.

**Table 6:** Adverse events within 72 h after surgery.

Variable	Group O (*n* = 50)	Group S (*n* = 50)	*p*-value
Dizziness	11 (22.00)	12 (24.00)	0.629
Respiratory depression	0(0.00)	0(0.00)	1.000
Hypotension	0(0.00)	0(0.00)	1.000
Urine retention	0(0.00)	0(0.00)	1.000
Constipation	0(0.00)	0(0.00)	1.000

Data are *n* (%).

## Discussion

Prior to the Phase III APOLLO trials (20, 21), it was demonstrated that oliceridine, a G protein-biased full agonist of the *μ*-opioid receptor, exhibits dose-dependent gastrointestinal adverse events. At low to moderate doses (0.1 and 0.35 mg), consistent with the biased agonism of oliceridine that minimizes *β*-arrestin recruitment, there is a significant reduction in the need for rescue antiemetic therapy in these dose groups, indicating a favorable gastrointestinal tolerability profile.

In this exploratory study, our findings indicated that the incidence of PONV within 72 h following bimaxillary orthognathic surgery was 50% in the control group. This result is slightly lower than the rate reported in our team’s previous investigation (5). Conversely, the 72-h PONV incidence was also 50% in the sufentanil group, a difference that did not achieve statistical significance (*p* = 0.315). This trend aligns with previous research (22), which documented a 48-h PONV incidence of 32.3% (21/65) in the oliceridine group compared to 50.8% (33/65) in the sufentanil group (OR = 0.46; 95% CI: 0.23–0.94; *p* = 0.033). While a numerically lower incidence was observed with oliceridine, these findings should be considered hypothesis-generating rather than confirmatory. Due to the limited sample size and the absence of *a priori* power calculation, this study lacks sufficient power to detect a clinically meaningful difference in PONV. Although the observed trends align with the biased agonism profile of oliceridine, they necessitate validation in larger, adequately powered trials.

In a previous VOLITION trial examining the efficacy of oliceridine for postoperative analgesia in patients undergoing non-cardiac surgery (24), the rate of complete gastrointestinal response (defined as 48 postoperative hours without vomiting or the need for rescue antiemetic therapy) was reported to be 53%. It is important to note that this endpoint did not account for nausea, a critical component of PONV, which may have led to an underestimation of the actual overall incidence of PONV. Despite this, the reported rate remains higher than the PONV incidence observed in our study. This discrepancy may be explained by variations in patient demographic and clinical characteristics between the two studies, as well as the possible implementation of more comprehensive perioperative antiemetic prophylaxis protocols at our institution.

The observed incidence of moderate-to-severe PONV within 72 h postoperatively was 26% in Group O and 36% in Group S. This difference did not reach statistical significance (*p* = 0.108), likely attributable to the limited sample size of this exploratory trial. Although a numerical reduction in moderate-to-severe episodes was noted with the use of oliceridine, these results should be considered hypothesis-generating only. Conversely, a recent randomized controlled trial conducted by Meng et al. (22) demonstrated a statistically significant reduction in moderate-to-severe PONV with oliceridine (18.5% vs. 38.5%, *p* = 0.012) in patients undergoing thoracoscopic surgery. The discrepancy between our findings and those of Meng et al. may be due to variations in surgical populations, baseline PONV risk, and the concurrent use of multimodal antiemetic strategies, which in our trial may have mitigated the differences between groups.

An analysis of the incidence of moderate-to-severe PONV across various postoperative intervals (0–6 h, 6–12 h, 12–24 h, 24–48 h, and 48–72 h) demonstrated that Group O (oliceridine) exhibited numerically lower rates during the 6–12 h and 12–24 h periods compared to Group S, as illustrated in Figure 2. This observation is consistent with the findings of Meng et al. (22), who reported a significantly lower 48-h incidence of PONV in the oliceridine group compared to the sufentanil group, with a more pronounced advantage observed within the initial 24 h. Although ANOVA analysis indicated that these differences did notachieve statistical significance, nor did the overall effect of group assignment on incidence over time, a significant temporal trend was identified (*F* = 6.706, *p* < 0.001). This trend confirms that the burden of moderate-to-severe PONV is greatest within the first 24 h post-surgery. This pattern aligns with our previous findings (5), underscoring the critical need to optimize prophylactic and management strategies specifically for the immediate postoperative period. Figure 3 illustrates that both groups attained similar levels of analgesic efficacy, as evidenced by a significant reduction in pain scores over time (*F* = 17.189, *p* < 0.001) and with no significant group-by-time interaction (*F* = 1.732, *p* = 0.142). The analogous trajectories reinforce the conclusion that the observed numerical differences in postoperative nausea and vomiting (PONV) were not attributable to variations in analgesic efficacy.

The analgesic protocol employed in this study was developed in alignment with our institution’s standard procedures for bimaxillary orthognathic surgery and based on the equianalgesic model established by the phase III Apollo trials (oliceridine:morphine ≈ 1:5; sufentanil:morphine ≈ 1:1,000). Both experimental groups demonstrated satisfactory analgesic outcomes, with no statistically significant differences identified between them. The pharmacokinetic profiles of oliceridine (25) reveal that, following a 1.5 mg dose, the area under the plasma concentration-time curve (AUC∞) maintains an intra-subject coefficient of variation (CV) of less than 20% across infusion durations ranging from 1 to 30 min. This suggests a consistent plasma concentration baseline, eliminating the necessity for dose adjustments based on infusion duration. Considering the potential influence of patient-specific variables, further large-scale dose-finding trials are warranted to determine the optimal PCIA dosage of oliceridine for bimaxillary orthognathic surgery.

Our multimodal antiemetic regimen, which includes dexamethasone and tropisetron, aligns with the current guidelines for the management of postoperative nausea and vomiting (26). Tropisetron, as a 5-HT_3_ receptor antagonist, generally exhibits greater efficacy in preventing vomiting than in alleviating nausea (27). The robust antiemetic properties of dexamethasone and tropisetron likely reduced the baseline incidence of moderate-to-severe PONV in both the control and intervention groups, thereby significantly diminishing the statistical power to detect any additional benefits from the intervention. This issue is corroborated by the findings of Labafchi et al. (28), who demonstrated that dexmedetomidine significantly decreased postoperative nausea (3.3% vs. 46.7%) when used alongside dexamethasone alone, without a 5-HT_3_ antagonist, following orthognathic surgery. In contrast, our study observed only a non-significant numerical reduction in PONV with oliceridine (40% vs. 50%). Consequently, while our regimen reflects real-world clinical practice for high-risk patients, it likely obscured the intrinsic antiemetic benefits of oliceridine.

The analysis revealed no significant differences in adverse events, specifically dizziness, between the two groups, and there were no instances of respiratory depression, hypotension, urinary retention, or constipation occurred in either cohort. The observed incidence of dizziness aligns with the findings reported by Meng et al. (22). Nonetheless, due to the limited sample size of the current study, further validation through larger-scale investigations may be necessary.

The current study is subject to several limitations. Firstly, the absence of an *a priori* sample size calculation, coupled with the small sample size, positions this study as exploratory; thus, the observed numerical reductions in PONV-related outcomes should be regarded as preliminary and hypothesis-generating. Secondly, the oliceridine PCIA regimen (0.25 mg/kg) was based on the equianalgesic model from phase III APOLLO trials (oliceridine:morphine ≈ 1:5; sufentanil:morphine ≈ 1:1000). The absence of a dose-finding design limited the exploration of dose-dependent effects on PONV and adverse events, leaving the optimal balance between analgesic efficacy and antiemetic benefits undetermined. Thirdly, the use of multimodal antiemetic prophylaxis, consistent with clinical practice, may have diminished potential differences between groups. Consequently, our findings do not provide definitive evidence of a superior antiemetic profile of oliceridine compared to sufentanil, but rather underscore the necessity for future dose-finding and confirmatory trials in populations at high risk for PONV.

## Conclusion

In this exploratory study, oliceridine demonstrated analgesic efficacy and safety comparable to that of sufentanil. Although numerical reductions in postoperative nausea and vomiting (PONV)-related outcomes were observed, they did not achieve statistical significance. These hypothesis-generating findings necessitate validation in larger, adequately powered trials employing dose-optimization designs.

## Data Availability

The raw data supporting the conclusions of this article will be made available by the authors, without undue reservation.
